# Supplementary material for: Comparative Analysis of Environment-Responsive Alternative Splicing in the Inflorescences of Cultivated and Wild Tomato Species
Source: Int J Mol Sci. 2022 Sep 30;23(19):11585. doi: 10.3390/ijms231911585 (PMC9569760; doi:10.3390/ijms231911585)
Supplement: Supplementary file 1 [file ijms-23-11585-s001.zip › Supplemental Figure S1 Putative proteins encoding by splice variants.pdf]

**Supplemental text and Figures**

**Supplemental text:** Putative protein isoforms encoding by splice variants of DAGs

**Supplemental Figure S1-S3**

Complete Go terms enriched in DAGs (Figure S1), DEGs (Figure S2) and DEIs (Figure S3).

**Supplemental text:**
**Putative protein isoforms encoding by splice variants of DAGs**

**Solyc01g091260 myeloid leukemia factor**
>TCONS_00010533=ITAG4.0_Solyc01g091260
MSKLFGGKNPFDDPFFTEPFGGWFGWNDPFDVQQDSRKQITIEELNPEGDGGQAQENSEPTKDLVVKNKKPSK
KSNGSQSFSYRRVSYGGLNGMYYICSEGKMIGPDGVVLAEMKEEDKTIGESLHTISKGIHNKGHSVTTKHSSD
GREDTLQTLHNLNEDELGDFEQNWKANADKYLPGWDKNFSLLENQGSISSLWDEFANWRGLGGYESPALEYYG
NAGPVAQVSESGEDSSRRATRRVPVE*
>TCONS_00010531/j1=TCONS_00010532/j2
MYYICSEGKMIGPDGVVLAEMKEEDKTIGESLHTISKGIHNKGHSVTTKHSSDGREDTLQTLHNLNEDELGDF
EQNWKANADKYLPGWDKNFSLLENQGSISSLWDEFANWRGLGGYESPALEYYGNAGPVAQVSESGEDSSRRAT
RRVPVE*

**Solyc01g099010 GDSL esterase/lipase**
>TCONS_00011617=ITAG4.0_Solyc01g099010
MAATIILLLTLLTVKIGDAEQQVLLKLKNPRLMNCRFDKIYQFGDSISDTGNCIRESLCKAQFWCKRPPYGMD
FYKHVTGRCSNGMLIIDFIAMECDLPLLNPYKDENAEFRHGVNFAVAGSTAISAEFLAENNIDNIGATNSSLS
VQLDWMSSHFHTTCSPNCPEKLNNSLFLVGEIGGNEFNFGFLQDKTIEESRKIVPEVVQTIIHSVKRIIGFGA
TRIIVPGNFPIGCIPFFLTKFMTNNSTAFDKHHCLKDLNNFVIFYNRYLQQAIDELKNDYPNITLIYGDYYNA
FMWLLENAVSLGFDNNSLQKACCGIGGEYNYNGHISCGDPMVPACVDPNTHISWDGIHFTQNAYSWLARWLID
DMLPKLNCQV*
>TCONS_00011615/j2=TCONS_00011614/j1
MNTGWMFLNPKPGFSSYLSCLFFHFRPFSSIKPPSFPVNSARGISLLLSTSGREGNVRLGSSIHASILKDPEL
VLLGNQYSTQDAFVVWNSLLTMYARCGRTQDASQVFDEMPVKDTVTWNSIVSGFLSNGNFKMGFGYFKQMMGS
DSLMFDHASVTTILSACDGLDFIMVNKMMHGLVLLSGLEREIAVSNALITSYFRCGCADSGRQVFDEMDMRNV
ISWTAVISGLAQNEFCEESLDLLVKMQNAAVVPNYLTYLSALLACSGMKALGEARQIHGIVWKLGFQSDLCIE
SALMDVYSKCGSVQDAWQMFESAGVLDTIAMTVMLVGFAKNGYEEEALQIFVKMVKAGVDIDPDVVSAILGVF
GSDTCLALGKQVHSLIIKKGFISNSFVRNGLINMYSKCGELEESVKIFNSIAQRNSVSWNSIIAAYARHGNGY
RTLQLYEEMRSYGVDPTDVTFISLLHACSHVGLVNKGMEFFESMQVIYGMTPRMEHYAAVVDLLGRAGLLCEA
KSFIEELPVKPDIFIWQALLGACSIHGDAEIGKYAADQWLLTSPDNPVPFVLLANIYSSRGRWKERARTIRKM
KETGVAKETGTSWIEIEKEIHCFVVADQMHPRTMIIYSTLLELFRHMRDEGYVPDNRFILYYMDGDETDFSAD
LPDTSELVGG*

**Solyc02g085420 U1 small nuclear ribonucleoprotein 70 kDa**
>TCONS_00023420=ITAG_Solyc02g085420=TCONS_00023418/j7=TCONS_00023419/j8
MGDFNDAFMRNNPNVQARAKAQNRANVMQLKLIGQSHPTGLTANLLKLFEPRPPLEYKPPPEKRKCPSYTGMA
QFVSNFAQPGDPEYAPPIPEVETPERRARIHKIRLEEGAKKAAEELEKYDPSSDPNATGDPYKTLFVARLNYE
TTESRVKREFEAYGPIKRVRLVMDKTNNKPRGYAFIEYVHTRDMKAAYKQADGKKIDNRRVLVDVERGRTVPN
WRPRRLGGGLGTTRVGNEDATREVVQPGRAASRSEEPRARDDRDRDREKSRERVRDKDREKSRERSHDRPRER
ERDDKHHRERERTREREKDRGRDRDRDRERDRTRDRERGKDRDRDGHRERDRHREKDRERGRDEGEVDQGRGR
SRDREYDYEHVDSKHERDRHGDKERNYDPAEPEDDHGHYDYYDHHQGRGDYENPDAQGGDDRYKDASRGHDRY
DQMEEDNYAYDHGASETKERDRDYKRSDRSHSREYDY*
>TCONS_00023417/j6
MGDFNDAFMRNNPNVQARAKAQNRANVMQLKLIGQSHPTGLTANLLKLFEPRPPLEYKPPPEKRKCPSYTGMA
QFVSNFAQPGDPEYAPPIPEVETPTERRARIHKIRLEEGAKKAAEELEKYDPSSDPNATGDPYKTLFVARLNY
ETTESRVKREFEAYGPIKRVRLVMDKTNNKPRGYAFIEYVHTRDMKA----
DGKKIDNRRVLVDVERGRTVPNWRPRRLGGGLGTTRVGNEDATREVVQPGRAASRSEEPRARDD
RDRDREKSRERVRDKDREKSRERSHDRPRERERDDKHHRERERTREREKDRGRDRDRDRERDRTRDRERGKDR
DRDGHRERDRHREKDRERGRDEGEVDQGRGRSRDREYDYEHVDSKHERDRHGDKERNYDPAEPEDDHGHYDYY
DHHQGRGDYENPDAQGGDDRYKDASRGHDRYDQMEEDNYAYDHGASETKERDRDYKRSDRSHSREYDY*
>TCONS_00023412/j1
MVKHLAWELFDSKVRLVMDKTNNKPRGYAFIEYVHTRDMKAAYKQADGKKIDNRRVLVDVERGRTVPNWRPRR
LGGGLGTTRVGNEDATKRWFSLEGQHLALRSQEHEMTGIGIGRSPVKGCEIRTERNLVSVLMTGQGNVKEMTN
IIGNAREPEKEKKIVVVTVIETANVIGHVTVSEAKTETGMVTVSEIVTVRRTGKEVGMKVRLTRVVGVLVIEN
MIMSMLIQNTSETDTVTKRGIMILQNLKMIMDIMTIMITTKVEEIMRIQMHKVGMIAIKMPVVVMIVMIKWKR
IIMLMTTGHLRQKKGIGITSVQIGHILVNMTTDDEEYASACVAVEDMAY*
>TCONS_00023413/j2=TCONS_00023415/j4

MVKHLAWELFDSKVRLVMDKTNNKPRGYAFIEYVHTRDMKAAYKQADGKKIDNRRVLVDVERGRTVPNWRPRR
LGGGLGTTRVGNEDATREVVQPGRAASRSEEPRARDDRDRDREKSRERVRDKDREKSRERSHDRPRERERDDK
HHRERERTREREKDRGRDRDRDRERDRTRDRERGKDRDRDGHRERDRHREKDRERGRDEGEVDQGRGRSRDRE
YDYEHVDSKHERDRHGDKERNYDPAEPEDDHGHYDYYDHHQGRGDYENPDAQGGDDRYKDASRGHDRYDQMEE
DNYAYDHGASETKERDRDYKRSDRSHSREYDY*
>TCONS_00023416/j5
MVKHLAWELFDSKVRLVMDKTNNKPRGYAFIEYVHTRDMKAAYKQADGKKIDNRRVLVDVERGRTVPNWRPRR
LGGGLGTTRVGNEDATREVVQPGRAASRSEEPRARDDRDRDREKSRERVRDKDREKSRERSHDRPRERERDDK
HHRERERTREREKDRGRDRDRDRERDRTRDRERGKDRDRDGHRERDRHREKDRERGRDEEPEDDHGHYDYYDH
HQGRGDYENPDAQGGDDRYKDASRGHDRYDQMEEDNYAYDHGASETKERDRDYKRSDRSHSREYDY*
>TCONS_00023414/j3
MSRAMMQYIFGLLFQATPEIDTCLYLVKKLLGVSTFSLLYSSYIPSMLLRFYFWIQRLSKKFQAFQETPAVMV
KHLAWELFDSKVRLVMDKTNNKPRGYAFIEYVHTRDMKAAYKQADGKKIDNRRVLVDVERGRTVPNWRPRRLG
GGLGTTRVGNEDATREVVQPGRAASRSEEPRARDDRDRDREKSRERVRDKDREKSRERSHDRPRERERDDKHH
RERERTREREKDRGRDRDRDRERDRTRDRERGKDRDRDGHRERDRHREKDRERGRDEGEVDQGRGRSRDREYD
YEHVDSKHERDRHGDKERNYDPAEPEDDHGHYDYYDHHQGRGDYENPDAQGGDDRYKDASRGHDRYDQMEEDN
YAYDHGASETKERDRDYKRSDRSHSREYDY*

**Solyc06g069020 Elongation factor**
>TCONS_00054263=ITAG4.0_ Solyc06g069020
MEEERKTDDVGKPDMPEDELRDEAHPDLKPEHVDPKGVSGKESSPPEDMQVEVEVNKKRHLNVVFIGHVDAGK
STIGGQILLLSGQVDDRTIQKYEKEAKDKNRESWYMAYIMDTNEEERVKGITVEVGRAHFETETTRFTILDAP
GHKSYVPNMISGASQADIGVLVISARKGEFETGYERGGQTREHVQLAKTLGVTKLIIVVNKMDDPTVNWSKER
YDEIESKMVPFLRSSGYNVKKDVQFLPISGLLGSNLKTRLEKSVCPWWSGHCLFEVLDAVEVPPRDPNGPLRM
PIIDKFKDMGTVVMGKIESGSIREGDNLLIMPNKAAVKVLAIFCDEDRVRHVGPGENVRVRLSGVEEDDLLSG
FVLCSVAKPIPAVTEFVAQLQILELLDNAIFTAGYKAVLHVHAVVEECEIVELMQQIDLKTKKPMKKKPLFVK
NGAIVLCRVQVNNMICVEKFSNFAQLGRFTLRTEGKTVAVGKITALPTVADSA*
>TCONS_00054260/j1=TCONS_00054261/j2
MDIEEDIKALQLDSSEDTVLVNVEDARPGEAIKHDKVDGEDSFMEEERKTDDVGKPDMPEDELRDEAHPDLKP
EHVDPKGVSGKESSPPEDMQVEVEVNKKRHLNVVFIGHVDAGKSTIGGQILLLSGQVDDRTIQKYEKEAKDKN
RESWYMAYIMDTNEEERVKGITVEVGRAHFETETTRFTILDAPGHKSYVPNMISGASQADIGVLVISARKGEF
ETGYERGGQTREHVQLAKTLGVTKLIIVVNKMDDPTVNWSKERYDEIESKMVPFLRSSGYNVKKDVQFLPISG
LLGSNLKTRLEKSVCPWWSGHCLFEVLDAVEVPPRDPNGPLRMPIIDKFKDMGTVVMGKIESGSIREGDNLLI
MPNKAAVKVLAIFCDEDRVRHVGPGENVRVRLSGVEEDDLLSGFVLCSVAKPIPAVTEFVAQLQILELLDNAI
FTAGYKAVLHVHAVVEECEIVELMQQIDLKTKKPMKKKPLFVKNGAIVLCRVQVNNMICVEKFSNFAQLGRFT
LRTEGKTVAVGKITALPTVADSA*
>TCONS_00054262/j3
MDIEEDIKALQLDSSEDTVLVNVEDARPGEAIKHDKVDGELRDEAHPDLKPEHVDPKGVSGKESSPPEDMQVE
VEVNKKRHLNVVFIGHVDAGKSTIGGQILLLSGQVDDRTIQKYEKEAKDKNRESWYMAYIMDTNEEERVKGIT
VEVGRAHFETETTRFTILDAPGHKSYVPNMISGASQADIGVLVISARKGEFETGYERGGQTREHVQLAKTLGV
TKLIIVVNKMDDPTVNWSKERYDEIESKMVPFLRSSGYNVKKDVQFLPISGLLGSNLKTRLEKSVCPWWSGHC
LFEVLDAVEVPPRDPNGPLRMPIIDKFKDMGTVVMGKIESGSIREGDNLLIMPNKAAVKVLAIFCDEDRVRHV
GPGENVRVRLSGVEEDDLLSGFVLCSVAKPIPAVTEFVAQLQILELLDNAIFTAGYKAVLHVHAVVEECEIVE
LMQQIDLKTKKPMKKKPLFVKNGAIVLCRVQVNNMICVEKFSNFAQLGRFTLRTEGKTVAVGKITALPTVADS
A*

**Solyc11g066830 Zinc finger transcription factor 68**
>TCONS_00093481=ITAG4.0_Solyc11g066830=TCONS_00093478/j1=TCONS_00093479/j
2=TCONS_00093480/j3
MAEHLASIFGTEKDRVNCPFYFKIGACRHGDRCSRLHTKPSISPTILLSNMYQRPDSITPGVDAQGNPIDPRK
IQEHFEDFYEDLFEELNKYGEIESLNICDNLADHMVGNVYVQFSEEEQAANALKNLTGRFYAGRPIIVDFSPV
TDFREATCRQYEENVCNRGGYCNFMHLKKISRELRRQLFGRYRTRHSRSRSRSPYRHRSYEDRSHRSHSRKHD
ERDHYYESRSRRNRSTSPDHRRGRSRSPGGRRDRSPVRDGSEERRARIEQWNREKEQAELDNRANADSNYKNE
SNENGSAPNQDQYYNQQ*

**Solyc01g105140 two different protein isoforms**

>TCONS_00012779=TCONS_00012775/j1=TCONS_00012776/j2=TCONS_00012777/j3
MSHFGRTGPPDIADTYSLLVLNITFRTSADDLFPLFDKYGKVVDIFIPRDRRTGESRGFAFVRYKYAEE
AQKAVDRLDGRVVDGREMAVQFAKYGPNAERIHQGRIIEKVPGFKGSSRSRSPRRRYRDDYHRDREYRR
SRSRSVDRYERDRYRQRERDYRHRSRSRSLSPDYDRDRGRRRDRKHYRRSPSVDSASPSRRSPSPHRKE
SPPRSLSPTKGSPVRRVRNERSPTPRSRSPPGRAMDSRSPSPRVDED*
>TCONS_00012778/j4
MISFLFSTSMGRTGESRGFAFVRYKYAEEAQKAVDRLDGRVVDGREMAVQFAKYGPNAERIHQGRIIEK
VPGFKGSSRSRSPRRRYRDDYHRDREYRRSRSRSVDRYERDRYRQRERDYRHRSRSRSLSPDYDRDRGR
RRDRKHYRRSPSVDSASPSRRSPSPHRKESPPRSLSPTKGSPVRRVRNERSPTPRSRSPPGRAMDSRSP
SPRVDED*

**Solyc02g061840 five different protein isoforms**

>TCONS_00020220
MADSPRQRYSRSPSPWEEKSRSGSRSRSRSPPGSYSRPKERSRSRSRSRSRGREEVSNPGNTLYVTGLS
TRVTERDLEEHFSKEGKVKSVFLVMEPRSRISRGFAFITMDSLEDANRCIKHLNQSVLEGRYITVEKSR
RKRARTPTPGHYLGLKNARDDGYRGDRGRYRDREDYGYRRSPRHSPYRGGRDYSPRRSPYGGRSRRERS
RSYSPYGRSYPRGPR*
>TCONS_00020217/j5=TCONS_00020219/j7
MADSPRQRYSRSPSPWEEKSRSGSRSRSRSPPGSYSRPKERSRSRSRSRSRGREEVSNPGNTLYVTGLS
TRVTERDLEEHFSKEGKVKSVFLVMEPRSRISRGFAFITMDSLEDANRCIKHLNQSVLEGRYITVEKKD
IASALLAATKDDQFFPCNISTQFDQSLAKTTFHMHLIN*
>TCONS_00020214/j2=TCONS_00020216/j4=TCONS_00020215/j3
MEPRSRISRGFAFITMDSLEDANRCIKHLNQSVLEGRYITVEKSRRKRARTPTPGHYLGLKNARDDGYR
GDRGRYRDREDYGYRRSPRHSPYRGGRDYSPRRSPYGGRSRRERSRSYSPYGRSYPRGPR*
>TCONS_00020218/j6
MLLQVPFRDPCRQSSHIKCVSRIRNFFPLPRLLDLDLDLDLSFGLEYDPGGDLDLDLDPDLDFSSQGEG
EREYLWRGESAISQDTRKP*
>TCONS_00020213/j1
MQGMMVIVETVAGIEIVRTMGTEDLQGIHHIEVDEIIHLGVRLMVEGQEGSVLGHILLMEGAILVVPDR
HKPQ*

**Solyc02g068440 two different protein isoforms**

>TCONS_00021035=TCONS_00021030/j1=TCONS_00021031/j2=TCONS_00021033/j4
MMLMCKGVWPSSSSVVLLQTPNGHKSFKTQASFSSYPLASKVMVRNLSYSTDESCLEKIFSNFGHVAEV
KIVKDEVTKRSKGYAFIQYTSQENAMLALDSMDHKYINGRVIFVELAKPTKKDFGRYPRSCGPPVERLP
SENEVPDLKENC*
>TCONS_00021032/j3=TCONS_00021034/j5
MDTNPSKPKLAFLATLLPAKLWLEIYHTPLMKVVWRKYFQILVMLLKVKIVKDEVTKRSKGYAFIQYTS
QENAMLALDSMDHKYINGRVIFVELAKPTKKDFGRYPRSCGPPVERLPSENEVPDLKENC*

**Solyc03g026240 three different protein isoforms**

>TCONS_00030837 =
MRPIFCGNFEFETRQPELERLFKRYGKVDRVDMKSGFAFVYMDDERDAKDAIQGLDRIEFGRKGRRLRV
EWSKEERSRKPEGSKKSSSSFRVSKTLFVINFDPYNTRTRDLERHFDPYGKILNIRIRRNFGFIQFETQ
EDATRALDATNMSKLMDRVITVEYAIRDDDDRKNGYGPGKTYNQSPRRGYDRGRSRSPRGRDRLSPDYG
RGRDRPSPDYGRGRDRPSPDYGRGRDRPISDFDRGRDRPNSDFGRGRDQLSPDYGRGPSRSPKHREGNS
EYGRGHSPAVGKERNPGHGNVRSPSPRRERTGPGNGLMSSPLNISPGYGDGPSPSAQRERRDKYSPDGH
NRGSSPGPKPEPVGSPVRDGRGSSE*
>TCONS_00030830/j2=TCONS_00030831/j3=TCONS_00030832/j4=TCONS_00030833
/j5=TCONS_00030834/j6=TCONS_00030835/j7=TCONS_00030836/j8
MDDERDAKDAIQGLDRIEFGRKGRRLRVEWSKEERSRKPEGSKKSSSSFRVSKTLFVINFDPYNTRTRD

LERHFDPYGKILNIRIRRNFGFIQFETQEDATRALDATNMSKLMDRVITVEYAIRDDDDRKNGYGPGKT
YNQSPRRGYDRGRSRSPRGRDRLSPDYGRGRDRPSPDYGRGRDRPSPDYGRGRDRPISDFDRGRDRPNS
DFGRGRDQLSPDYGRGPSRSPKHREGNSEYGRGHSPAVGKERNPGHGNVRSPSPRRERTGPGNGLMSSP
LNISPGYGDGPSPSAQRERRDKYSPDGHNRGSSPGPKPEPVGSPVRDGRGSSE*
>TCONS_00030829/j1
MSKLMDRVITVEYAIRDDDDRKNGYGPGKTYNQSPRRGYDRGRSRSPRGRDRLSPDYGRGRDRPSPDYG
RGRDRPSPDYGRGRDRPISDFDRGRDRPNSDFGRGRDQLSPDYGRGPSRSPKHREGNSEYGRGHSPAVG
KERNPGHGNVRSPSPRRERTGPGNGLMSSPLNISPGYGDGPSPSAQRERRDKYSPDGHNRGSSPGPKPE
PVGSPVRDGRGSSE*

**Solyc05g007200 two different protein isoforms**
>TCONS_00048162=TCONS_00048161/j2
MQKNQLFYLLQHTYTAIFYYRGAHQQKILHIYGTEYKGTSIAVFIPTPFSHTFHLNTSEADMNPGGYTV
EVTGLSPAATEKDVQEFFAFCGAIEHVEIVRAGEHASTAYVTFRNPHALETAVLLSGAAILDQRVCITS
WGHYQDDFDYWNHSSWKPQEDCHSSDSQGHHFVSSAGEAVTLTQDVVKTMLSQGYVLGKGALGKAKAFD
ESHGLSATAVSKVADLSERIGLTDKFCAGVEVARSVDQRYHISDTTRSAVSATGRTAISAATAVINSSY
FSKGALWMSGALSKAAQAAADLGSRGTNK*
>TCONS_00048160/j1
MNPGGYTVEVTGLSPAATEKDVQEFFAFCGAIEHVEIVRAGEHASTAYVTFRNPHALETAVLLSGAAIL
DQRVCITSWGHYQDDFDYWNHSSWKPQEDCHSSDSQGHHFVSSAGEAVTLTQDVVKTMLSQGYVLGKGA
LGKAKAFDESHGLSATAVSKVADLSERIGLTDKFCAGVEVARSVDQRYHISDTTRSAVSATGRTAISAA
TAVINSSYFSKGALWMSGALSKAAQAAADLGSRGTNK*

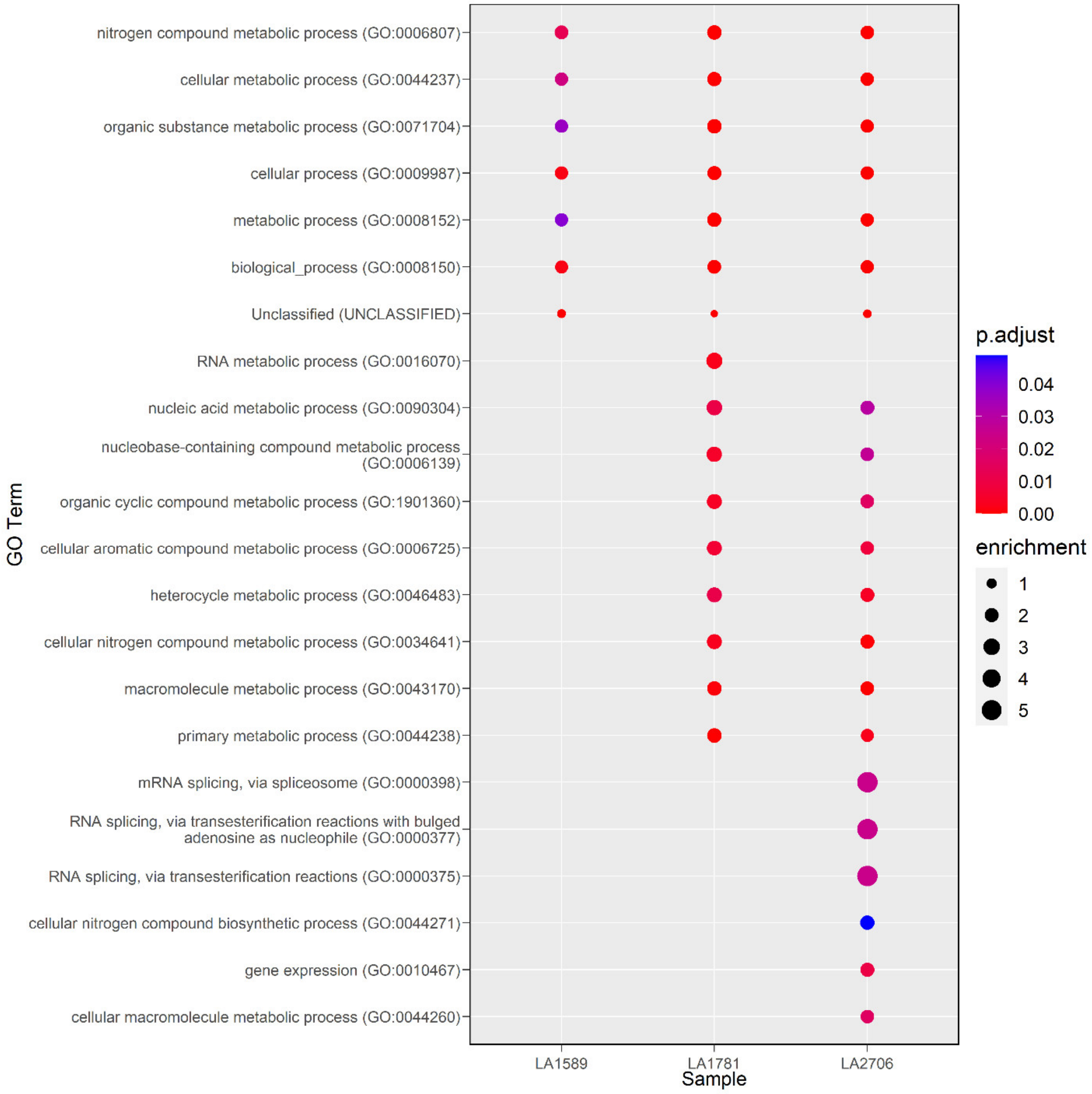


**Figure S1. Enriched GO terms in DAGs**
All enriched GO terms were included.

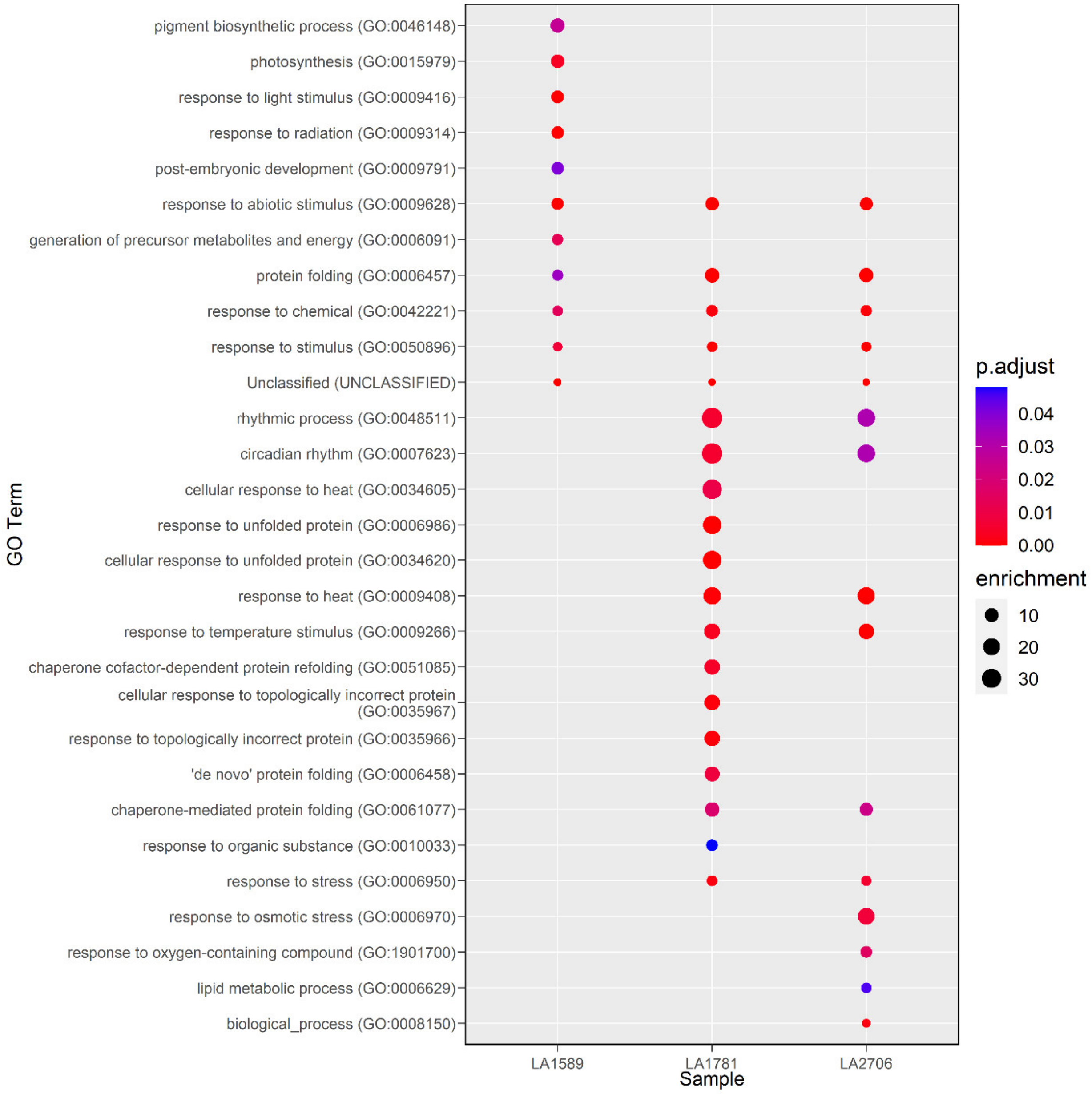


**Figure S2. Enriched GO terms in DEGs**

All enriched GO terms were included.

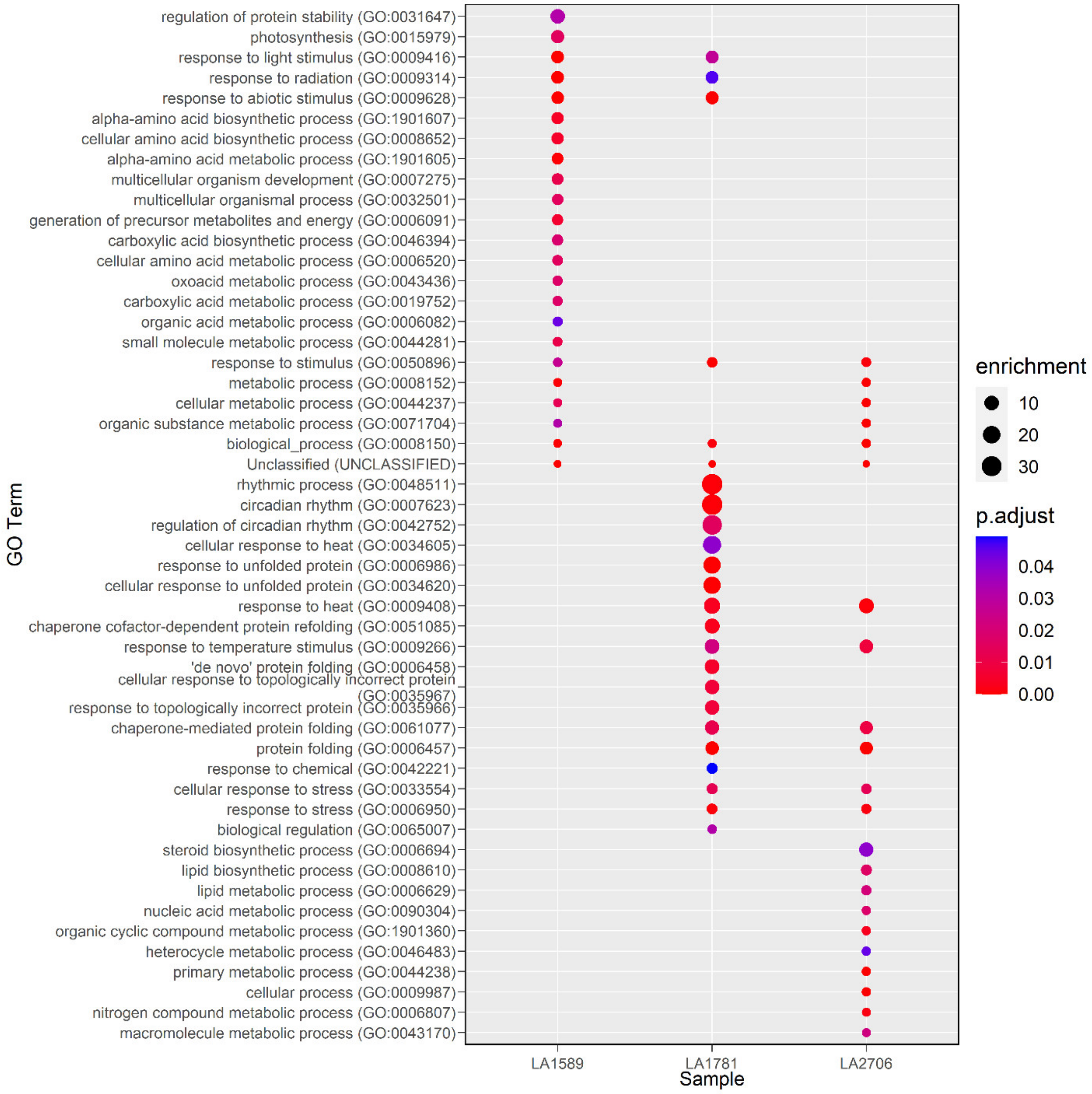


**Figure S3. Enriched GO terms in DEIs**
All enriched GO terms were included.
